# “Hunting” for the pseudoaneurysm in a vascular maze: Endoscopic ultrasound solving the puzzle

**DOI:** 10.1055/a-2106-0907

**Published:** 2023-06-27

**Authors:** Jayanta Samanta, Jahnvi Dhar, Uday Kiran Mangipudi, Bikkina Venkat Siddharda, Pankaj Gupta, Vikas Gupta

**Affiliations:** 1 Department of Gastroenterology, Post Graduate Institute of Medical Education and Research, Chandigarh, India; 2 Department of Radiodiagnosis and Imaging, Post Graduate Institute of Medical Education and Research, Chandigarh, India; 3 Department of GI Surgery, Post Graduate Institute of Medical Education and Research, Chandigarh, India

A 43-year-old man with chronic calcific pancreatitis presented with melena for 15 days. He received four units of a packed red blood cell transfusion before presenting at our center. On evaluation, investigations revealed anemia (hemoglobin 5.6 gm/dl) with tachycardia. After initial resuscitation, the patient was taken for esophagogastroduodenoscopy, which revealed no gastric or duodenal varices or any non-variceal source of bleeding. Computed tomography angiography (CTA) [arterial phase] revealed a contrast-filled bi-lobed outpouching from the gastroduodenal artery (GDA) suggestive of a pseudoaneurysm (size 1 × 1.2 cm) ( Fig. 1 ). On the venous phase, the portal vein was partially thrombosed with multiple collaterals surrounding the pseudoaneurysm ( Fig. 2 ).

**Fig. 1:**
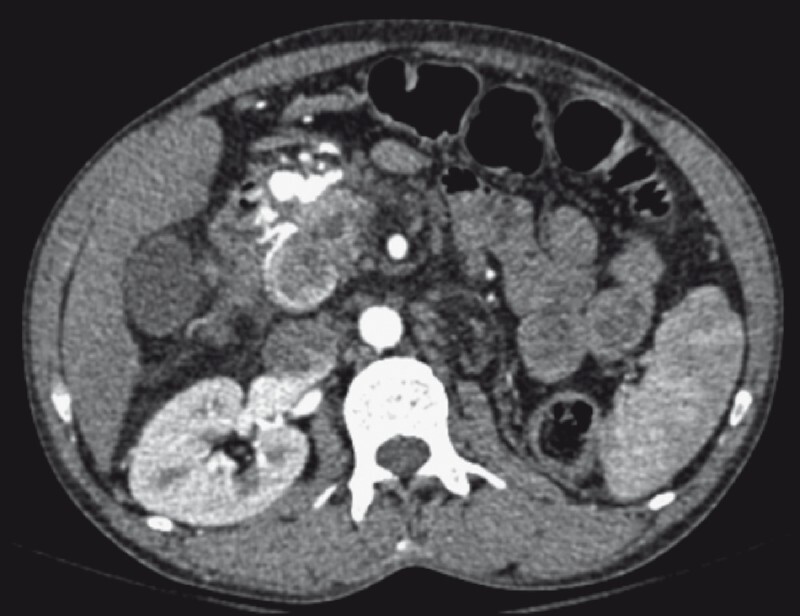
Computed tomography angiography (CTA) (arterial phase) revealed a contrast-filled bi-lobed outpouching from the gastroduodenal artery with no active contrast leak, suggestive of a pseudoaneurysm.

**Fig. 2:**
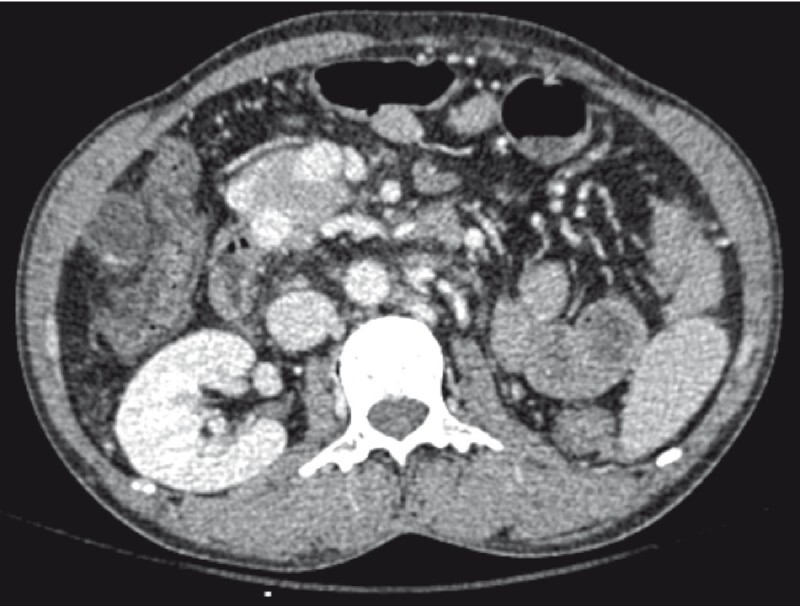
CTA (venous phase) revealed multiple collaterals surrounding the gastroduodenal artery pseudoaneurysm forming a vascular maze.

Because he was a poor candidate for radiological or surgical intervention, endoscopic ultrasound (EUS)-guided angioembolization was planned ( Video 1 ). EUS-guided localization of the pseudoaneurysm was done. In view of the multiple collaterals in a crowded peri-pancreatic location, it was difficult to identify the pseudoaneurysm up front. Thus, power Doppler was used sequentially for each dilated vascular channel to map the vessels by their flow pattern. This was done until the pseudoaneurysm could be localized. Once that was accomplished and confirmed by the arterial flow pattern on power Doppler ( Fig. 3 ), the pseudoaneurysm was punctured using a 19-G needle (EZ Shot3 Plus; Olympus Medical, Tokyo, Japan), aspiration of blood was performed to confirm the position, and subsequently one Nester coil (8 mm × 7 cm) was deployed ( Fig. 4 ) followed by 2 ml of cyanoacrylate-glue injection, leading to complete obliteration. A follow-up EUS and CTA 48 hours later showed complete obliteration with no flow and a patent GDA ( Fig. 5 ). At the 9-month follow-up, the patient was doing fine with no further bleeding episodes.

**Video 1** ‘Hunting’ of the pseudoaneurysm in the vascular maze using endoscopic ultrasound (EUS) with color-Doppler followed by EUS-guided angioembolization using coil-glue for complete obliteration.


**Fig. 3:**
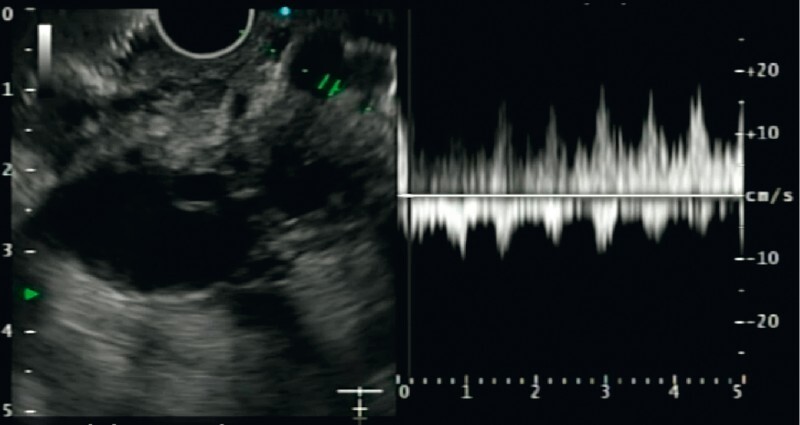
Endoscopic ultrasound (EUS)-guided localization of the gastroduodenal artery pseudoaneurysm in the vascular maze using a power Doppler showing arterial flow pattern.

**Fig. 4:**
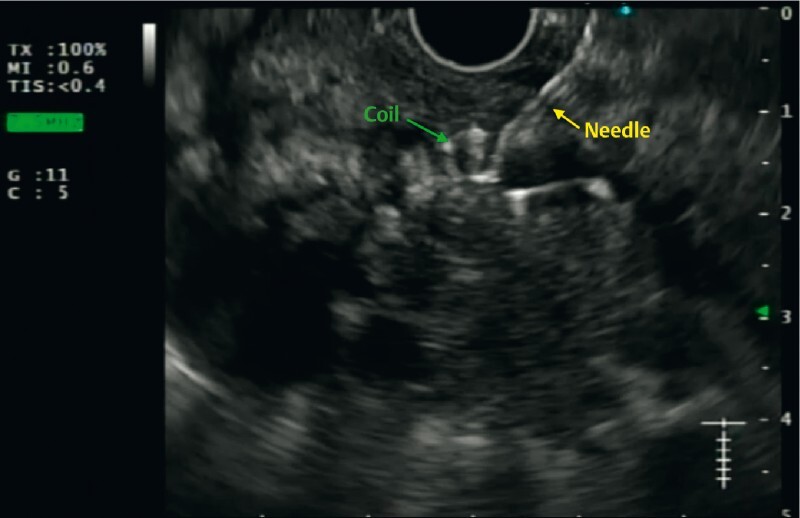
EUS-guided puncture of the pseudoaneurysm and deployment of coil.

**Fig. 5:**
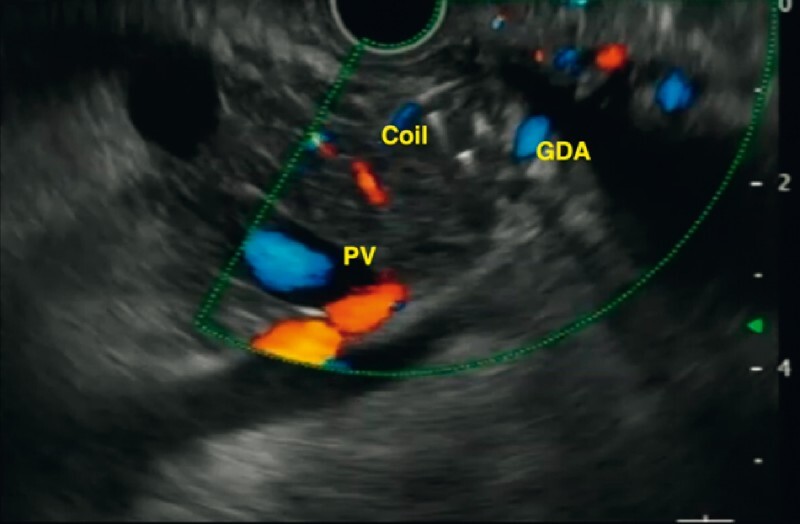
Follow-up EUS revealed completely obliterated pseudoaneurysm (with coil-glue complex), with no flow on Doppler and a patent gastroduodenal artery.

Co-existence of venous and arterial abnormalities within the same anatomical field is rare and can pose significant therapeutic challenge. EUS-guided angio-therapy with power Doppler can be an effective option in complex situations in which radiological or surgical intervention is difficult.

Endoscopy_UCTN_Code_TTT_1AS_2AG

## Notes

Endoscopy E-Videos

https://eref.thieme.de/e-videos
*E-Videos* is an open access online section of the journal *Endoscopy* , reporting on interesting cases and new techniques in gastroenterological endoscopy. All papers include a high-quality video and are published with a Creative Commons CC-BY license. Endoscopy E-Videos qualify for HINARI discounts and waivers and eligibility is automatically checked during the submission process. We grant 100% waivers to articles whose corresponding authors are based in Group A countries and 50% waivers to those who are based in Group B countries as classified by Research4Life (see: https://www.research4life.org/access/eligibility/ ). This section has its own submission website at https://mc.manuscriptcentral.com/e-videos

